# Supplementary figures and images for: Differential interaction patterns of opioid analgesics with µ opioid receptors correlate with ligand-specific voltage sensitivity
Source: eLife. 2023 Nov 20;12:e91291. doi: 10.7554/eLife.91291 (PMC10849675; doi:10.7554/eLife.91291)

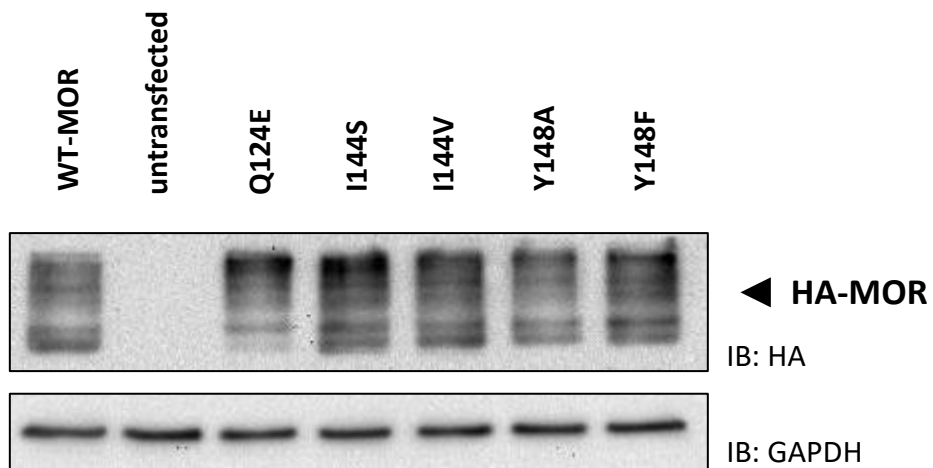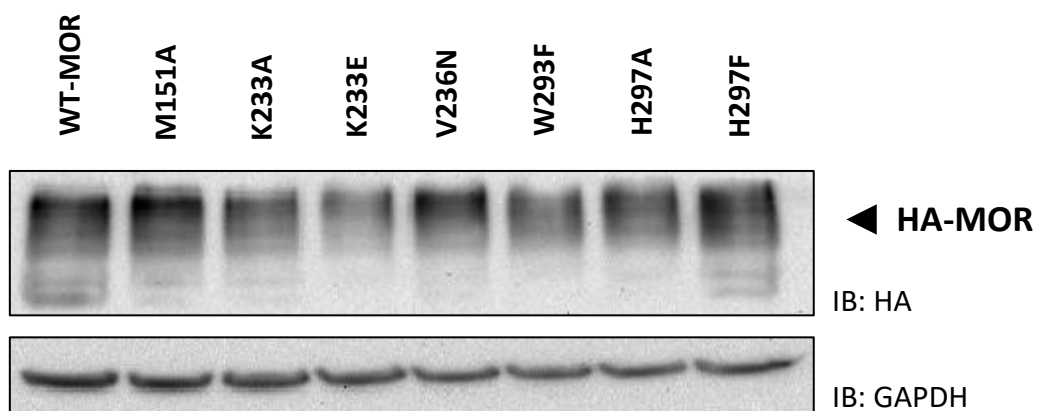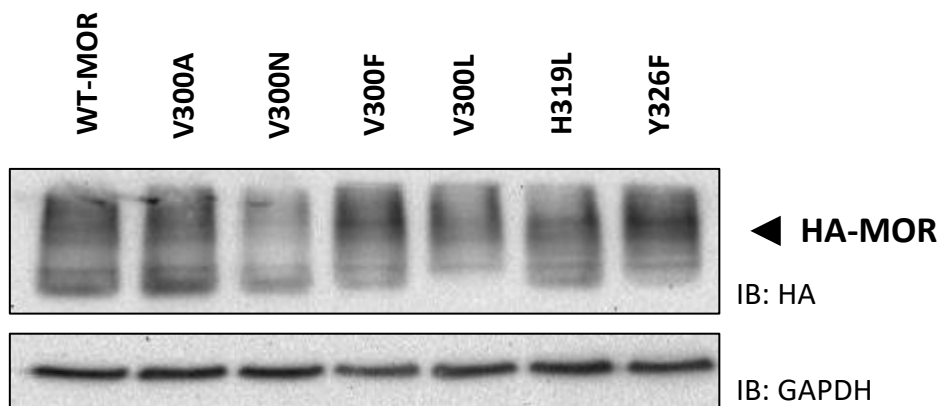

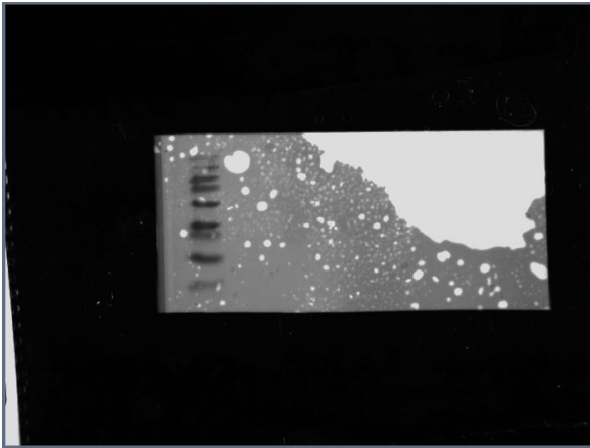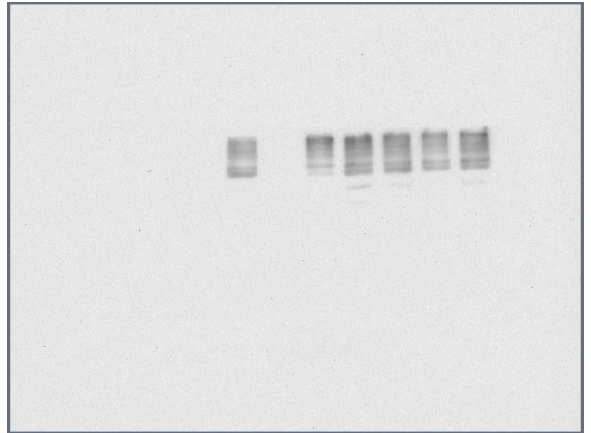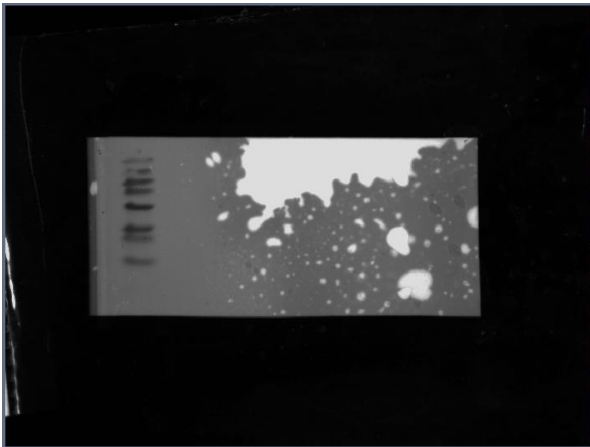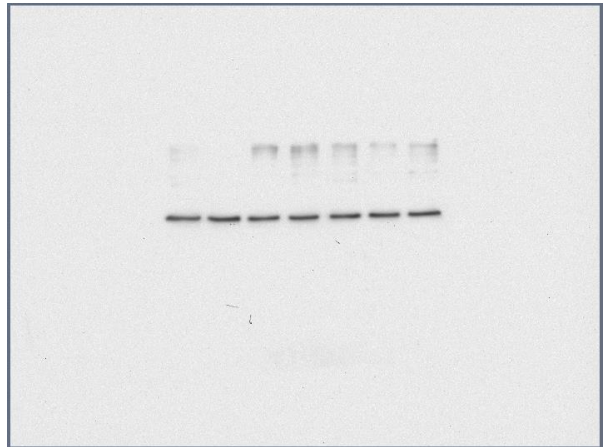

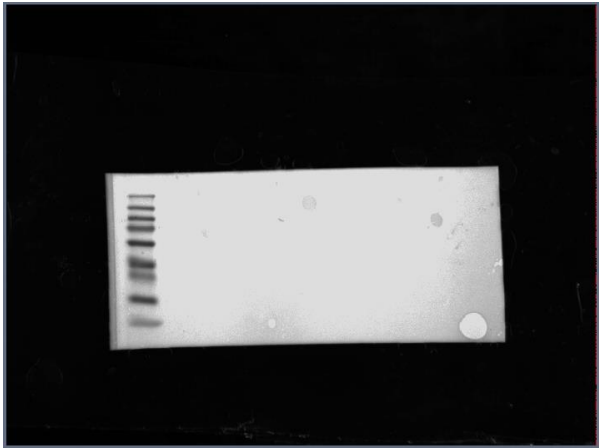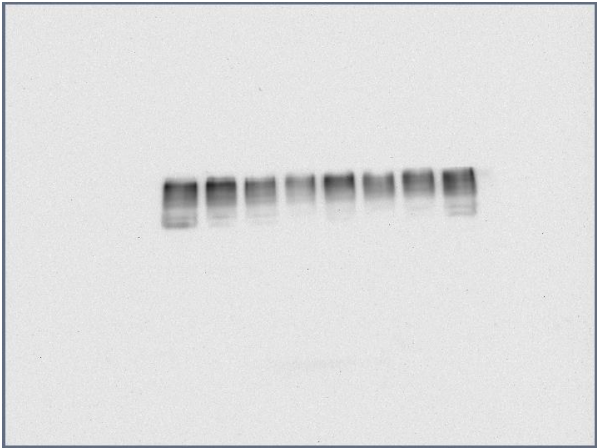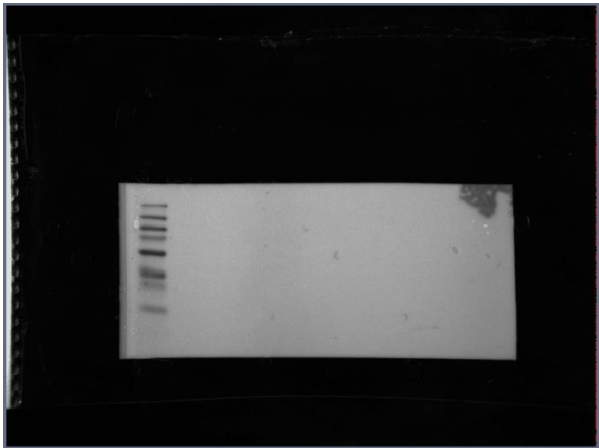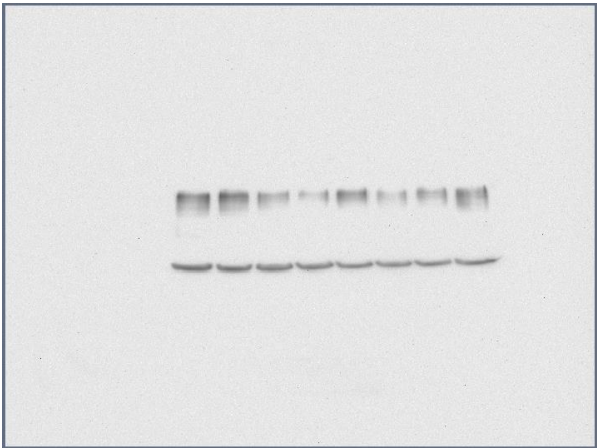

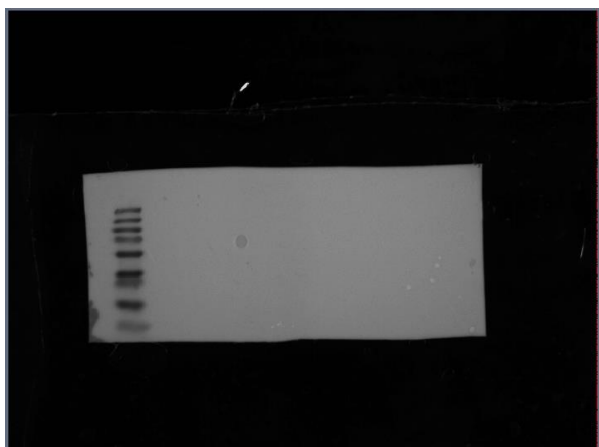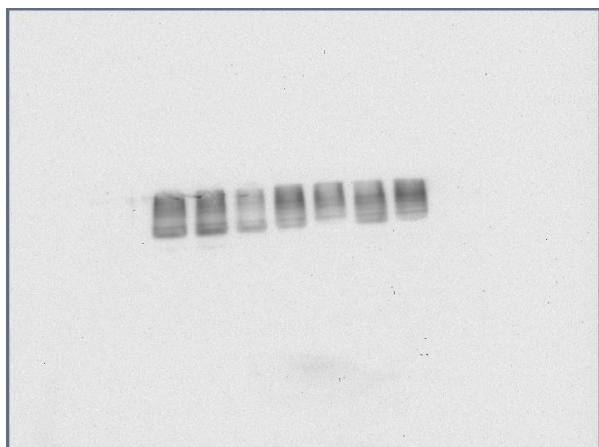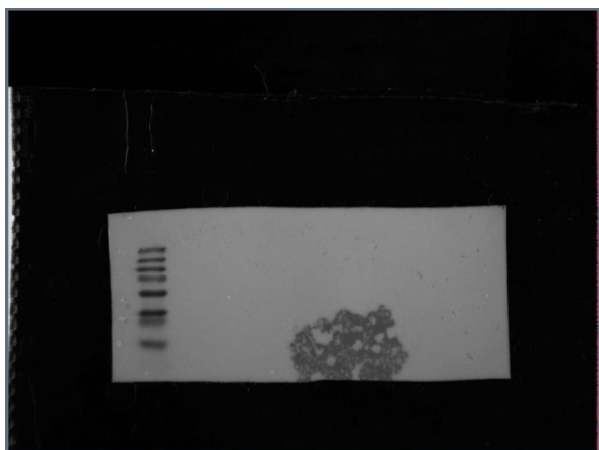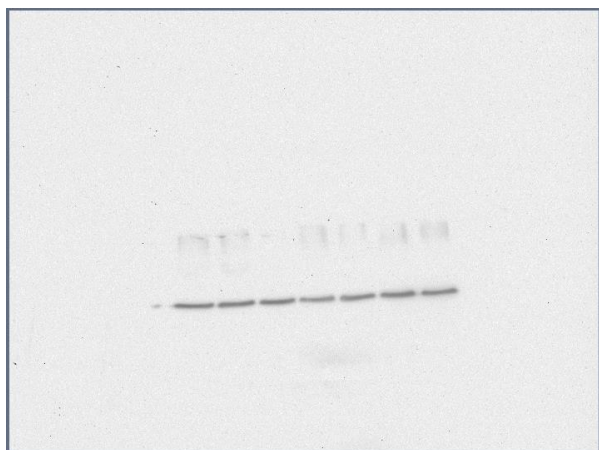

Supplement: Figure 3—figure supplement 4—source data 1. [file elife-91291-fig3-figsupp4-data1.pdf]

33.1

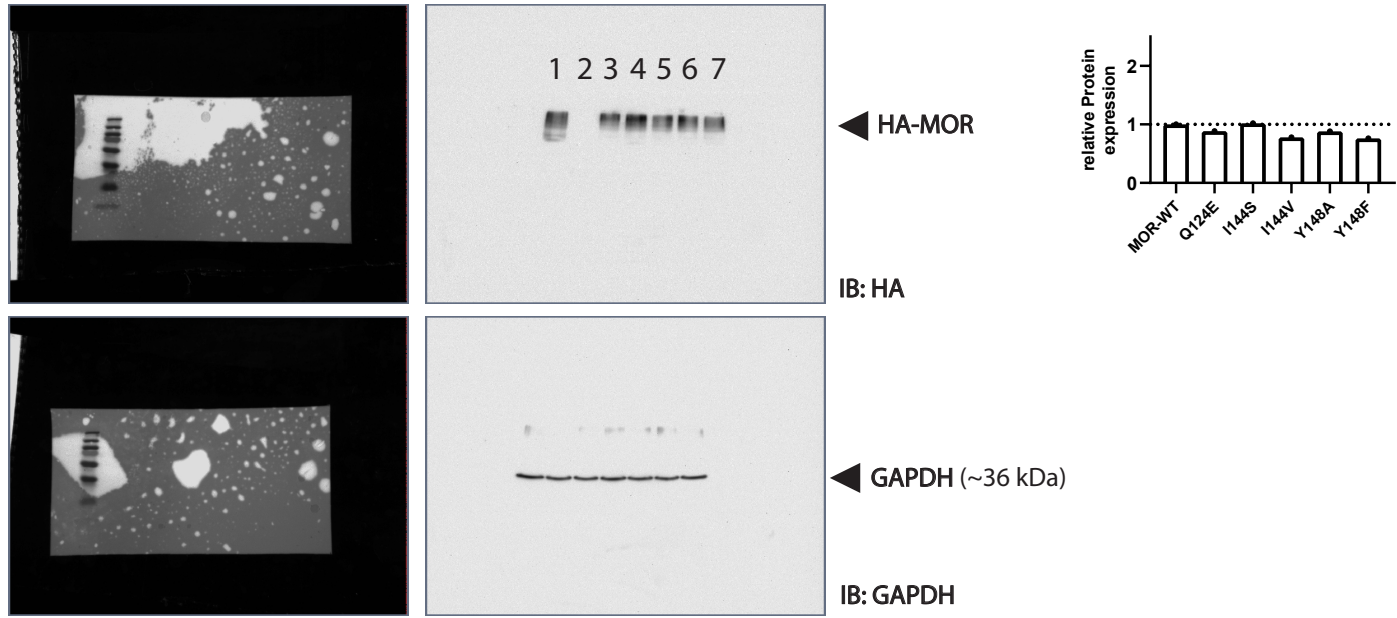

34.1

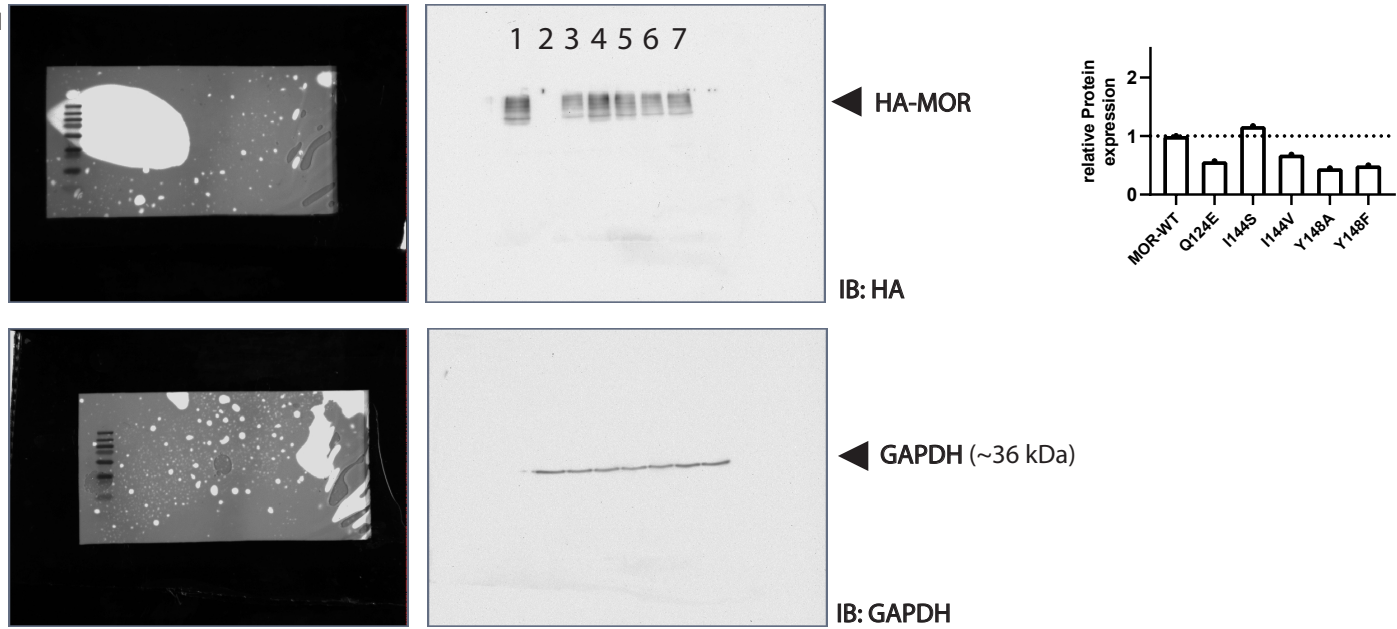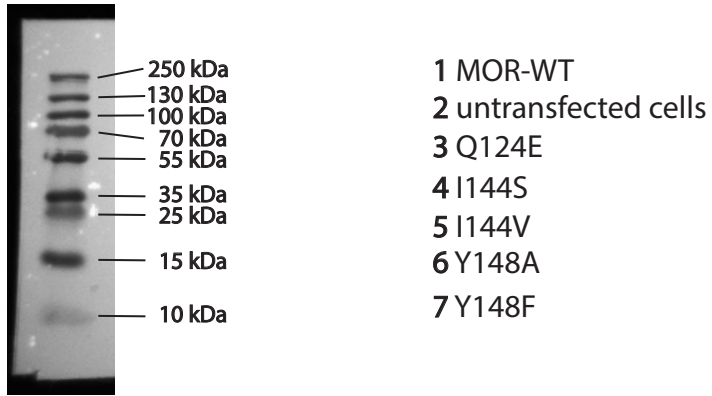

Supplement: Figure 3—figure supplement 4—source data 2. [file elife-91291-fig3-figsupp4-data2.pdf]

34.3

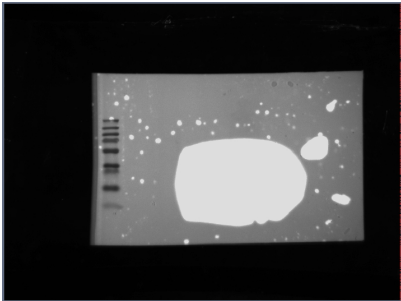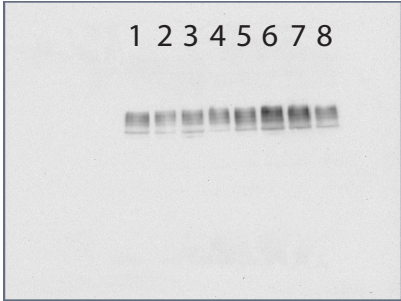

◀ HA-MOR

IB: HA

◀ GAPDH (~36 kDa)

IB: GAPDH

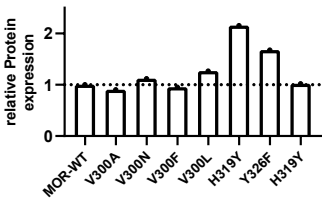

37.1

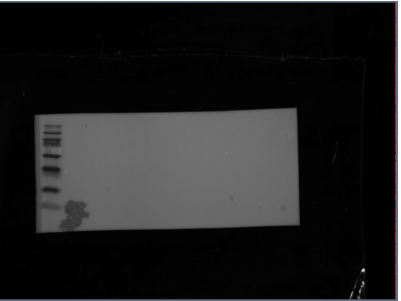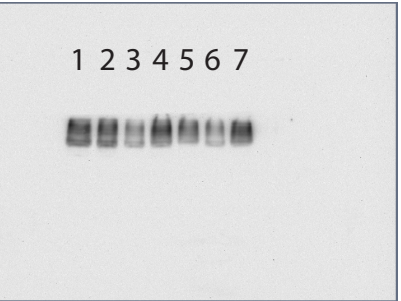

◀ HA-MOR

IB: HA

◀ GAPDH (~36 kDa)

IB: GAPDH

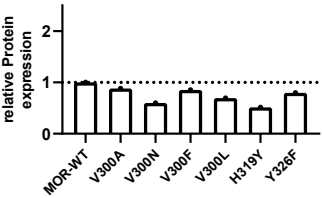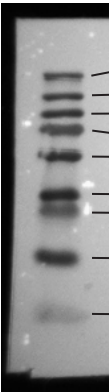

- 1 MOR-WT
- 2 V300A
- 3 V300N
- 4 V300F
- 5 V300L
- 6 H319Y
- 7 Y326F
- 8 H319Y

Supplement: Figure 3—figure supplement 4—source data 4. [file elife-91291-fig3-figsupp4-data4.pdf]

35.1

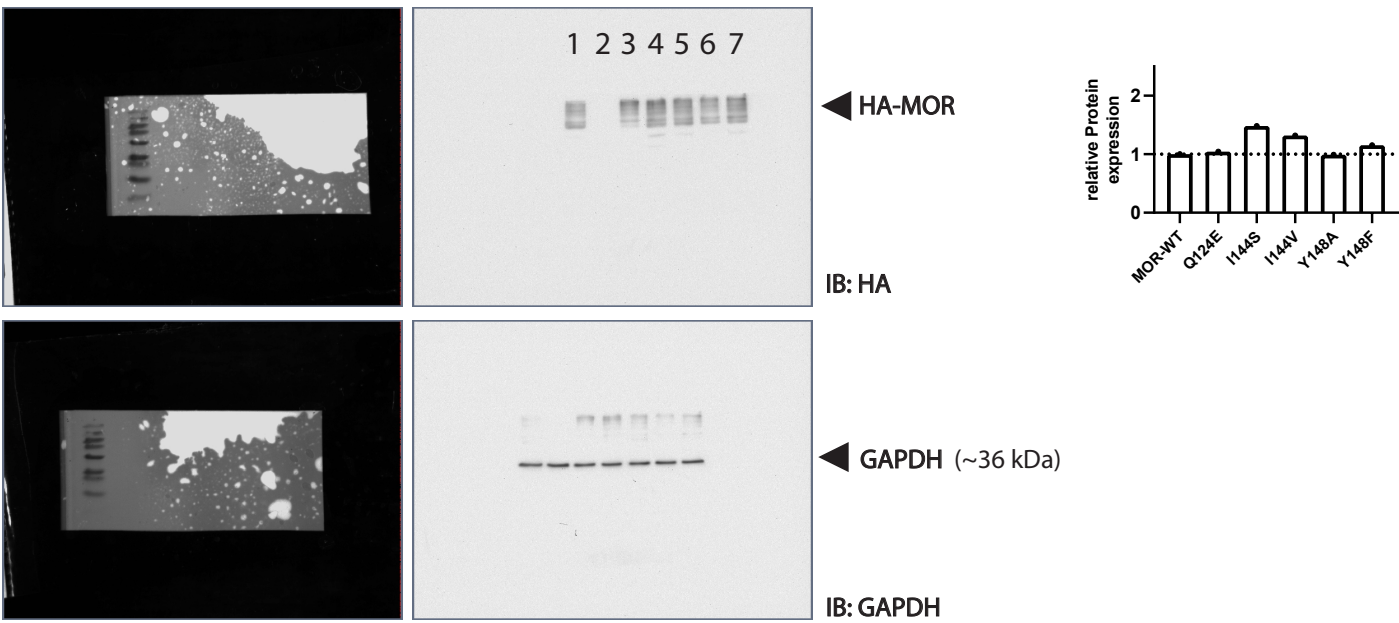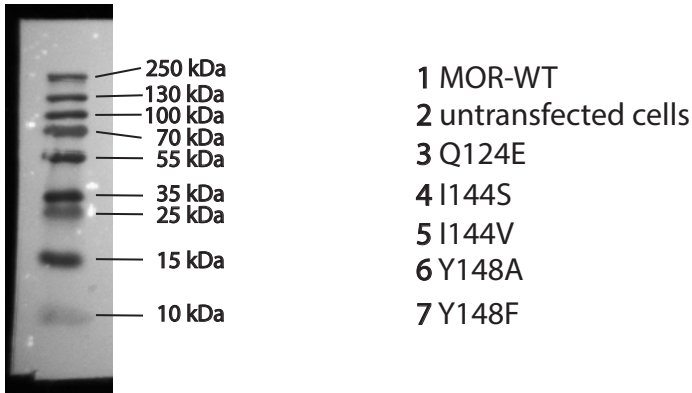

Supplement: Figure 3—figure supplement 4—source data 5. [file elife-91291-fig3-figsupp4-data5.pdf]

36 2.1

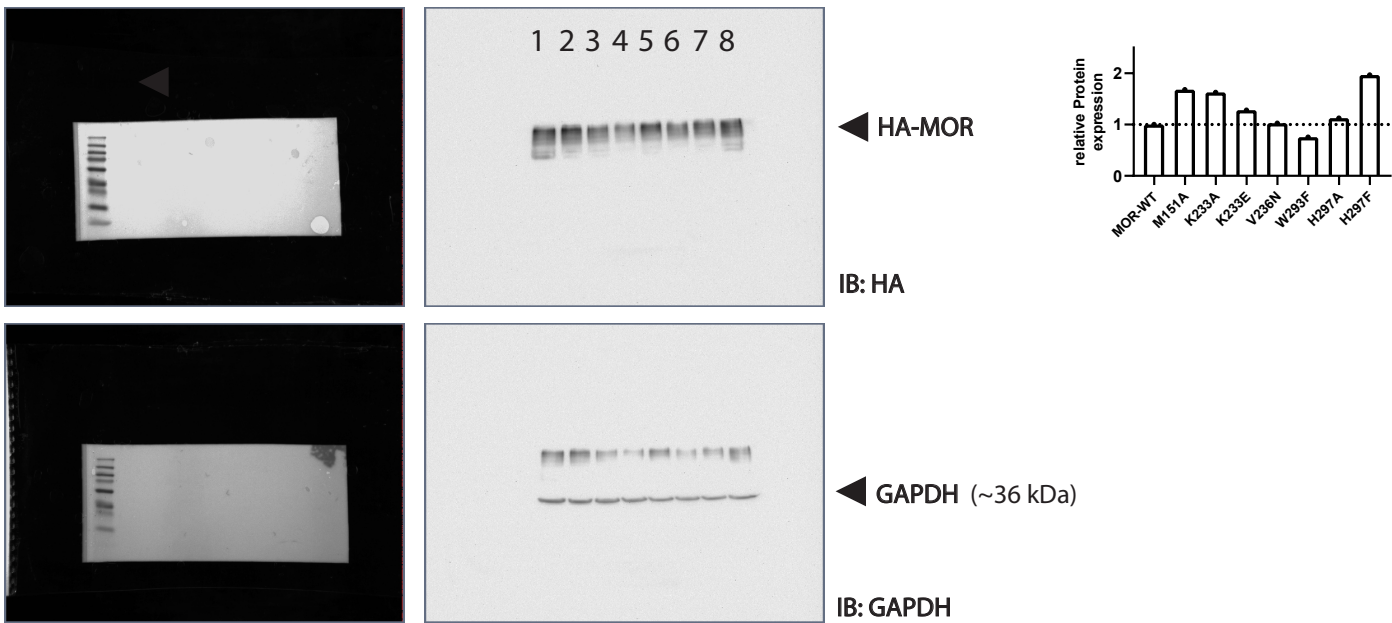

37.2

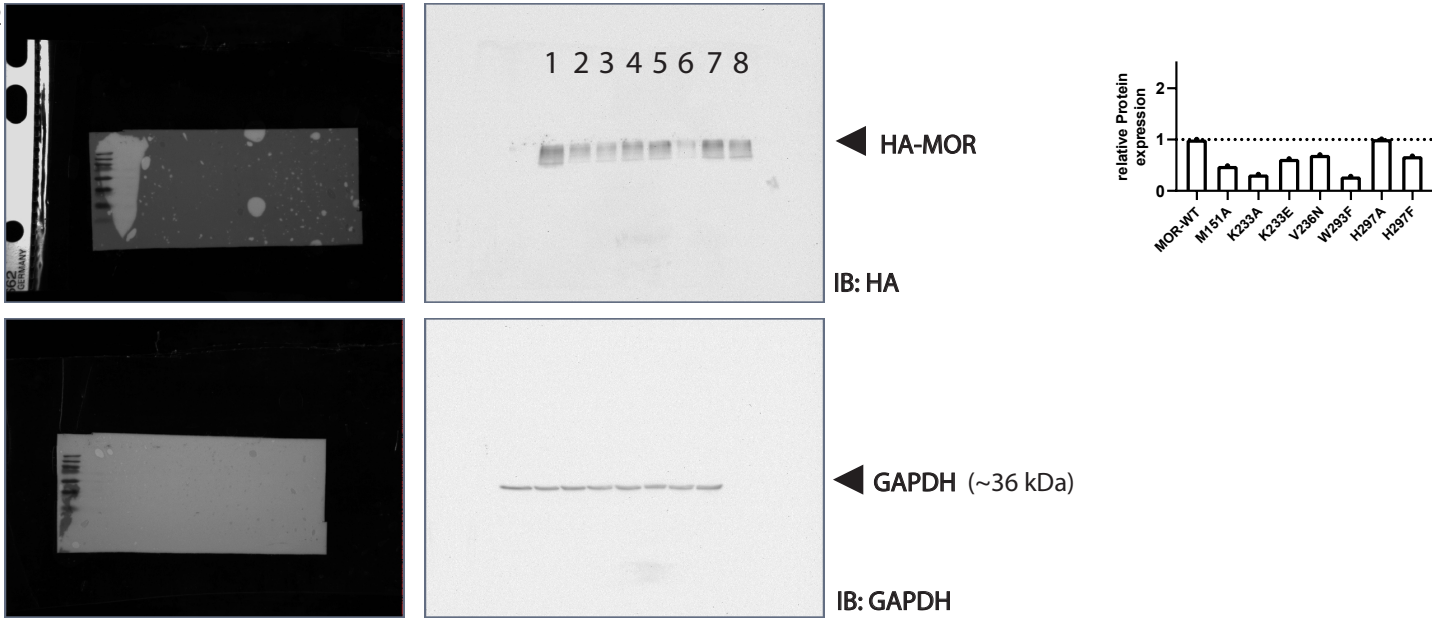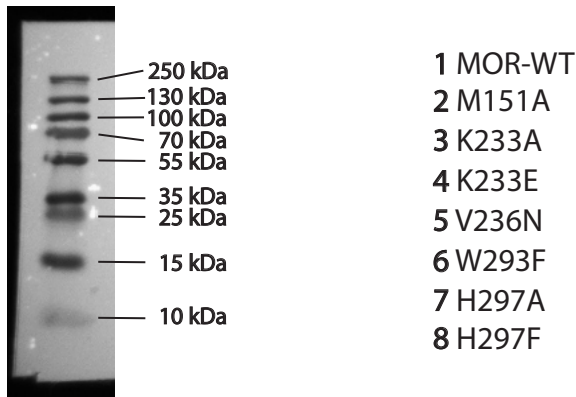

Supplement: Figure 3—figure supplement 4—source data 6. [file elife-91291-fig3-figsupp4-data6.pdf]

37.3

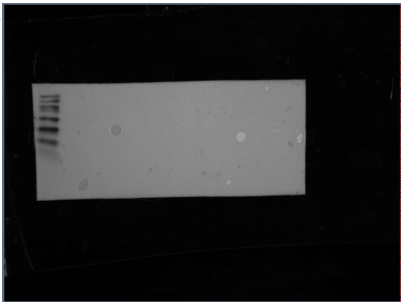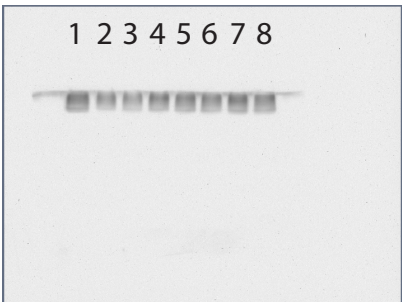

◀ HA-MOR

IB: HA

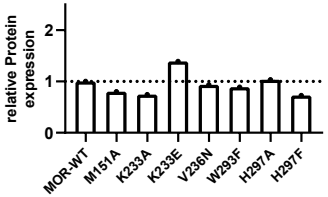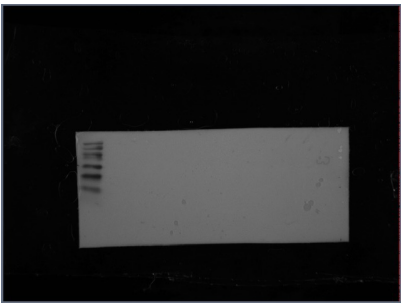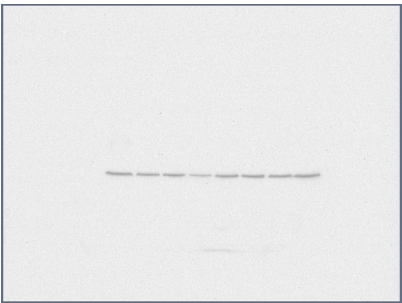

◀ GAPDH (~36 kDa)

IB: GAPDH

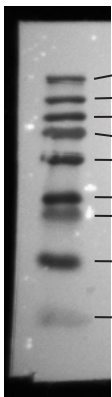

250 kDa  
130 kDa  
100 kDa  
70 kDa  
55 kDa  
35 kDa  
25 kDa  
15 kDa  
10 kDa

1 MOR-WT  
2 M151A  
3 K233A  
4 K233E  
5 V236N  
6 W293F  
7 H297A  
8 H297F

Supplement: Figure 3—figure supplement 4—source data 7. [file elife-91291-fig3-figsupp4-data7.pdf]
