# Supplementary material for: Differential interaction patterns of opioid analgesics with µ opioid receptors correlate with ligand-specific voltage sensitivity
Source: eLife. 2023 Nov 20;12:e91291. doi: 10.7554/eLife.91291 (PMC10849675; doi:10.7554/eLife.91291)
Supplement: Figure 3—figure supplement 4—source data 3. [file elife-91291-fig3-figsupp4-data3.pdf]

34.2

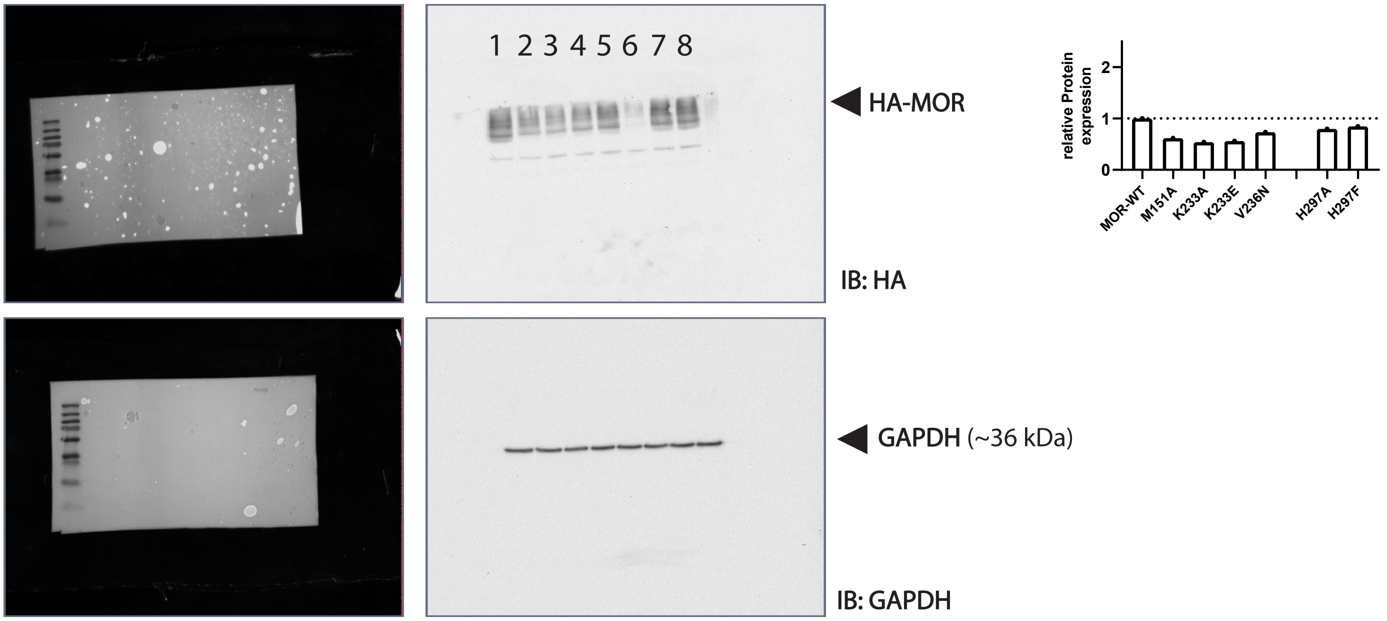

35.2

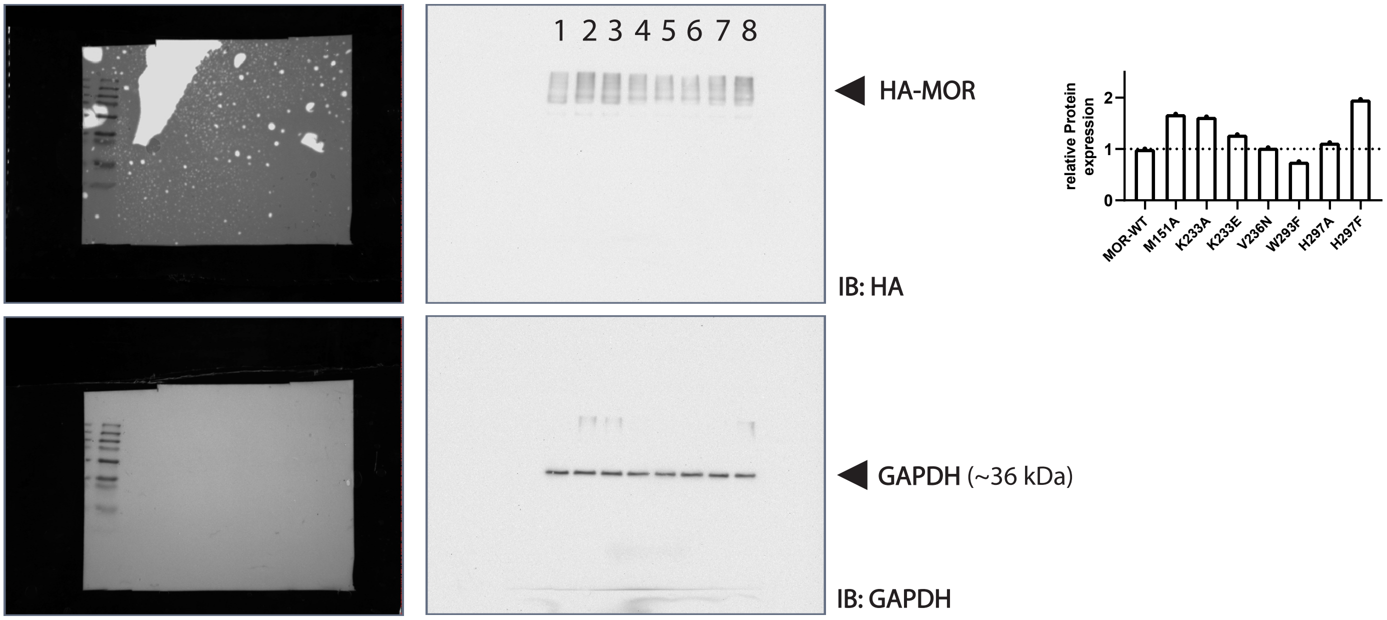

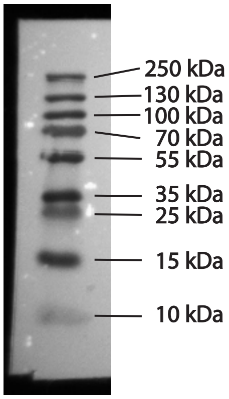

1 MOR-WT
2 M151A
3 K233A
4 K233E
5 V236N
6 W293F
7 H297A
8 H297F
