# Supplementary material for: Differential interaction patterns of opioid analgesics with µ opioid receptors correlate with ligand-specific voltage sensitivity
Source: eLife. 2023 Nov 20;12:e91291. doi: 10.7554/eLife.91291 (PMC10849675; doi:10.7554/eLife.91291)
Supplement: Figure 3—figure supplement 4—source data 8. [file elife-91291-fig3-figsupp4-data8.pdf]

37.4

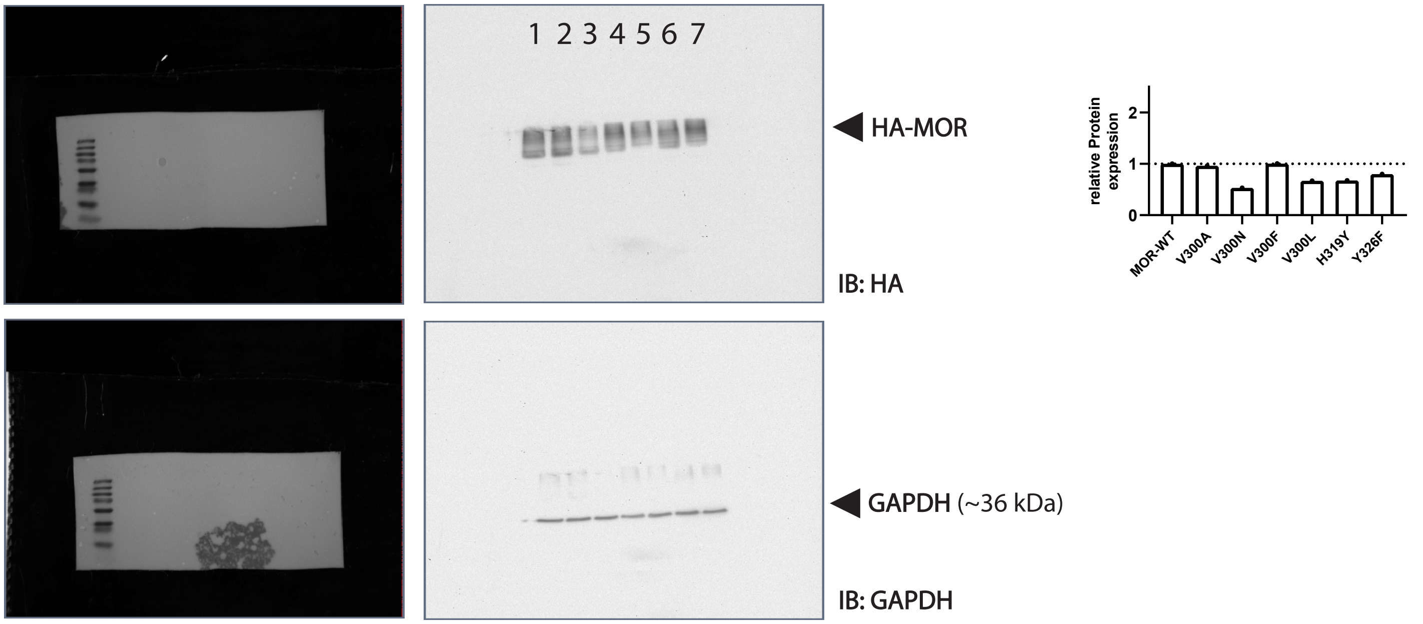

36.3

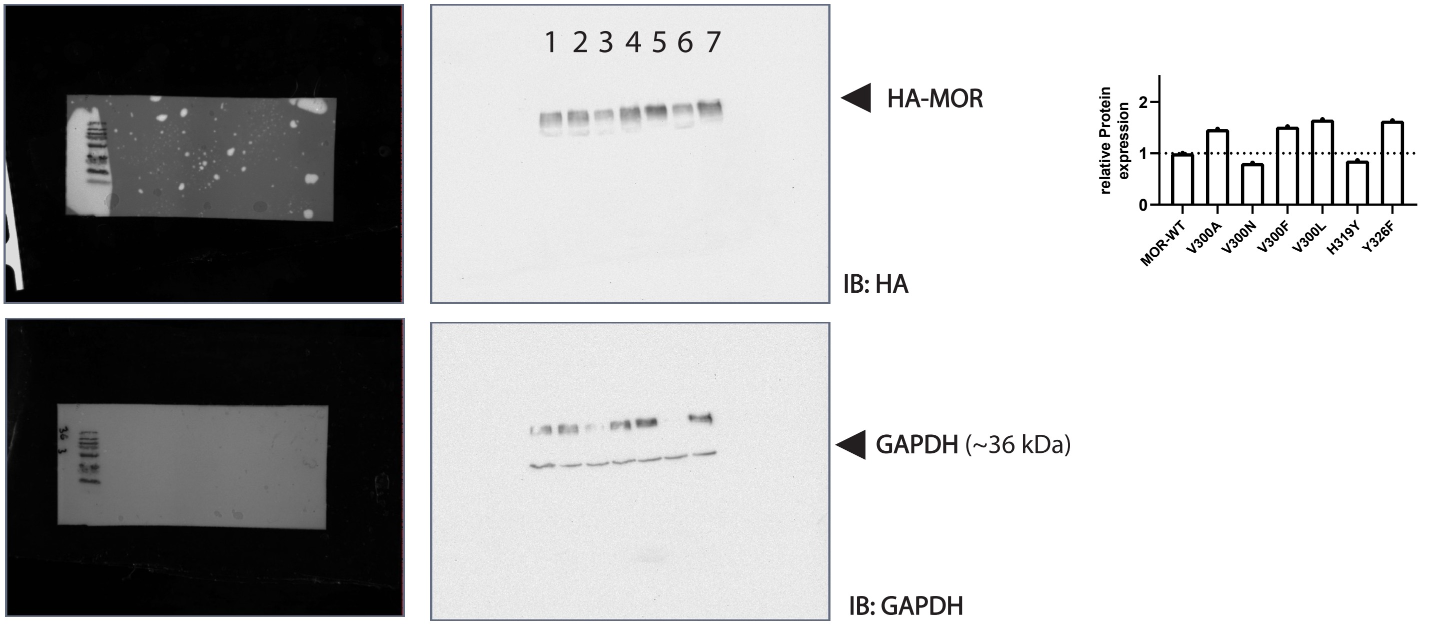

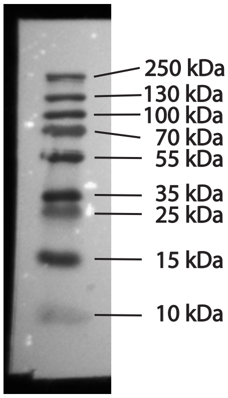

1 MOR-WT
2 V300A
3 V300N
4 V300F
5 V300L
6 H319Y
7 Y326F
