# Supplementary material for: Differential interaction patterns of opioid analgesics with µ opioid receptors correlate with ligand-specific voltage sensitivity
Source: eLife. 2023 Nov 20;12:e91291. doi: 10.7554/eLife.91291 (PMC10849675; doi:10.7554/eLife.91291)
Supplement: Supplementary file 1. [file elife-91291-supp1.docx]

**Supplementary File 1: Ligand properties;** 2D structures were taken from wikipedia

| **Ligand** | **TPSA** | **MW** | **HAcceptor** | **HDonor** | **Hetero**  **Atoms** | **Rotatable**  **bond** | **CrippenlogP** |
| --- | --- | --- | --- | --- | --- | --- | --- |
| Buprenorphine  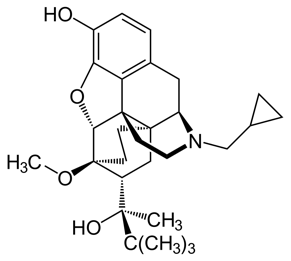 | 63,36 | 468,658 | 4 | 3 | 5 | 4 | 2,9967 |
| Etorphine  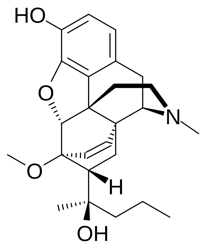 | 63,36 | 412,55 | 4 | 3 | 5 | 4 | 1,7465 |
| Fentanyl  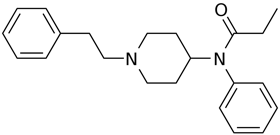 | 24,75 | 337,487 | 1 | 1 | 3 | 6 | 2,7196 |
| Loperamide  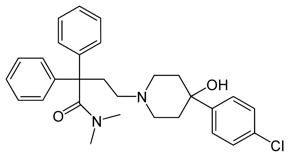 | 44,98 | 478,056 | 2 | 2 | 5 | 7 | 3,6709 |
| Meptazinol  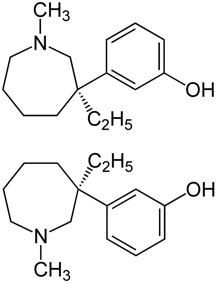 | 24,67 | 234,363 | 1 | 2 | 2 | 2 | 1,7386 |
| Methadone  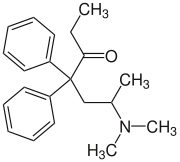 | 21,51 | 310,461 | 1 | 1 | 2 | 7 | 2,8749 |
| Morphine  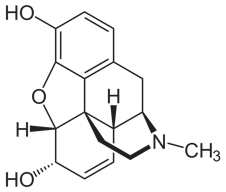 | 54,13 | 286,351 | 3 | 3 | 4 | 0 | -0,219 |
| Naloxone  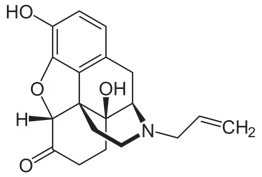 | 71,2 | 328,388 | 4 | 3 | 5 | 2 | -0,1157 |
| Pethidine  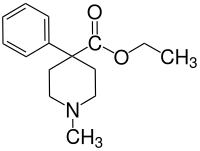 | 30,74 | 248,346 | 2 | 1 | 3 | 3 | 0,796 |
| PZM21  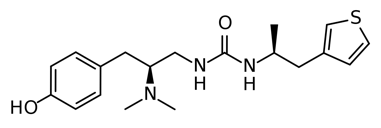 | 65,8 | 362,519 | 3 | 4 | 6 | 8 | 1,4397 |
| SR17018  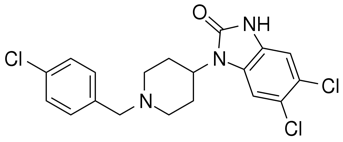 | 42,23 | 411,74 | 2 | 2 | 7 | 3 | 3,7098 |
| Tramadol  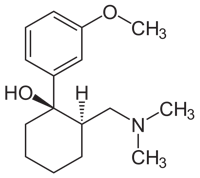 | 33,9 | 264,389 | 2 | 2 | 3 | 4 | 1,2175 |
| TRV130  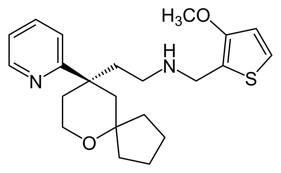 | 47,96 | 387,569 | 4 | 1 | 5 | 7 | 3,6664 |
| DAMGO  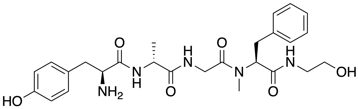 | 175,71 | 514,603 | 6 | 6 | 11 | 13 | -1,6557 |
