# Supplementary material for: Differential interaction patterns of opioid analgesics with µ opioid receptors correlate with ligand-specific voltage sensitivity
Source: eLife. 2023 Nov 20;12:e91291. doi: 10.7554/eLife.91291 (PMC10849675; doi:10.7554/eLife.91291)
Supplement: Supplementary file 2. [file elife-91291-supp2.docx]

**Supplementary File 2: Calculated pEC50 values for G protein activation and pIC50 values for fluorescent ligand binding competition**

|  | **Morphine** |  |  |  | **Methadone** |  |  |  | **Fentanyl** |  |  |  |
| --- | --- | --- | --- | --- | --- | --- | --- | --- | --- | --- | --- | --- |
|  | **pEC50** | **95%CI pEC50** | **pIC50** | **95%CI pIC50** | **pEC50** | **95%CI pEC50** | **pIC50** | **95%CI pIC50** | **pEC50** | **95%CI pEC50** | **pIC50** | **95%CI pIC50** |
| **WT** | 9.35 | 9.24-9.40 | 6.35 | 4.68-8.03 | 6.62 | 6.52-6.76 | 9.10 | 7.74-10.46 | 9.51 | 9.31-9.91 | 7.66 | 6.48-8.85 |
| **Q124E** | 6.54 | 6.28-7.34 | 4.15 | 1.20-7.09 | 6.41 | 6.05-∝ | 8.22 | 7.11-9.33 | 6.82 | 6.61-7.23 | 7.90 | 6.37-9.43 |
| **I144S** | 8.99 | 8.66-9.38 | 6.92 | 5.24-8.61 | 7.83 | 7.73-∝ | 8.22 | 7.08-9.36 | 7.89 | 7.61-8.17 | 8.12 | 6.89-9.36 |
| **I144V** | 8.01 | 7.54-8.51 | 5.11 | 3.37-6.84 | 7.52 | 7.33-7.70 | 8.16 | 6.28-9.60 | 8.49 | 7.90-9.66 | 7.04 | 6.25-7.83 |
| **Y148A** | 4.55 | 4.32-4.72 | 6.98 | 5.21-8.75 | 5.71 | 5.36-6.20 | 8.72 | 7.67-9.77 | 6.78 | 6.55-7.06 | 6.71 | 5.43-7.98 |
| **Y148F** | 6.14 | 5.89-6.43 | 4.97 | 0.51-9.43 | 5.85 | 5.76-∝ | 8.48 | 6.34-10.61 | 6.68 | 6.47-6.90 | 8.56 | 7.22-9.89 |
| **M151A** | 9.52 | 8.94-11.69 | x | x | 9.08 | 8.62-9.62 | x | x | 8.81 | 8.48-9.39 | x | x |
| **K233A** | 7.11 | 6.96-7.28 | x | x | 6.8 | 6.54-7.10 | x | x | 7.63 | 7.39-7.87 | x | x |
| **K233E** | 6.76 | 6.55-6.99 | x | x | 9.23 | 8.78-9.82 | x | x | 7.49 | 7.26-7.72 | x | x |
| **V236N** | 3.38 | 0.93-4.68 | 4.42 | 1.01-7.76 | 5.81 | 5.52-6.04 | 6.91 | 3.89-9.93 | 6.93 | 6.67-7.20 | 8.59 | 6.75-10.44 |
| **W293F** | 6.78 | 6.35-7.27 | x | x | 8.68 | 8.28-9.13 | x | x | x | x | x | x |
| **H297A** | 4.73 | 4.51-5.21 | x | x | 3.64 | 3.47-3.95 | x | x | 5.71 | 5.54-6.00 | x | x |
| **H297F** | 5.83 | 5.45-6.29 | x | x | 5.11 | 4.60-5.58 | x | x | 6.36 | 5.99-6.82 | x | x |
| **V300A** | 7.65 | 7.40-7.90 | 4.64 | 3.64-5.82 | 6.35 | 6.05-6.66 | 6.84 | 4.38-9.30 | 7.58 | 7.22-8.05 | 5.00 | -∝-6.92 |
| **V300N** | 5.72 | 5.53-∝ | 6.54 | 4.93-8.15 | 6.31 | 6.16-6.46 | 8.94 | 6.73-11.14 | 6.8 | 6.62-6.98 | 9.81 | 5.37-14.25 |
| **V300F** | 6.76 | 6.55-7.00 | x | x | 5.69 | 5.25-6.24 | x | x | 5.55 | 5.39-5.83 | x | x |
| **V300L** | 5.52 | 5.37-5.70 | x | x | 5.66 | 5.46-5.88 | x | x | 7.11 | 6.88-7.34 | x | x |
| **H319Y** | 6.94 | 6.39-8.02 | 4.44 | 2.65-6.23 | 5.92 | 5.83-6.00 | 7.08 | 5.07-9.10 | 7.05 | 6.71-7.60 | 5.80 | 4.56-7.04 |
| **Y326F** | 5.65 | 5.41-5.97 | 6.01 | 3.97-8.05 | 4.91 | 4.73-5.12 | 6.77 | 4.77-8.78 | 5.52 | 5.34-5.71 | 6.95 | 3.99-9.91 |
